# On the Evolution of the Cardiac Pacemaker

**DOI:** 10.3390/jcdd4020004

**Published:** 2017-04-27

**Authors:** Silja Burkhard, Vincent van Eif, Laurence Garric, Vincent M. Christoffels, Jeroen Bakkers

**Affiliations:** 1 Hubrecht Institute-KNAW and University Medical Center Utrecht, 3584 CT Utrecht, The Netherlands; s.burkhard@hubrecht.eu (S.B.); l.garric@hubrecht.eu (L.G.); 2 Department of Medical Biology, Academic Medical Center Amsterdam, 1105 AZ Amsterdam, The Netherlands; v.w.w.vaneif@amc.uva.nl (V.v.E.); v.m.christoffels@amc.uva.nl (V.M.C.); 3 Department of Medical Physiology, Division of Heart and Lungs, University Medical Center Utrecht, 3584 CT Utrecht, The Netherlands

**Keywords:** pacemaker cell, heart evolution, sinoatrial node, zebrafish, heart development

## Abstract

The rhythmic contraction of the heart is initiated and controlled by an intrinsic pacemaker system. Cardiac contractions commence at very early embryonic stages and coordination remains crucial for survival. The underlying molecular mechanisms of pacemaker cell development and function are still not fully understood. Heart form and function show high evolutionary conservation. Even in simple contractile cardiac tubes in primitive invertebrates, cardiac function is controlled by intrinsic, autonomous pacemaker cells. Understanding the evolutionary origin and development of cardiac pacemaker cells will help us outline the important pathways and factors involved. Key patterning factors, such as the homeodomain transcription factors Nkx2.5 and Shox2, and the LIM-homeodomain transcription factor Islet-1, components of the T-box (Tbx), and bone morphogenic protein (Bmp) families are well conserved. Here we compare the dominant pacemaking systems in various organisms with respect to the underlying molecular regulation. Comparative analysis of the pathways involved in patterning the pacemaker domain in an evolutionary context might help us outline a common fundamental pacemaker cell gene programme. Special focus is given to pacemaker development in zebrafish, an extensively used model for vertebrate development. Finally, we conclude with a summary of highly conserved key factors in pacemaker cell development and function.

## 1. Introduction

The heart is an evolutionary success story. During the course of evolution, novel structures and functions have been added to the primitive ancient pump. The network of transcription factors regulating mammalian embryonic heart development shows a high degree of evolutionary conservation. Similar signalling pathways controlling muscle growth, patterning, and contractility have been found in animals as distantly related as humans and drosophila [1,2,3,4,5,6,7,8,9,10,11,12,13].

**Figure 1:**
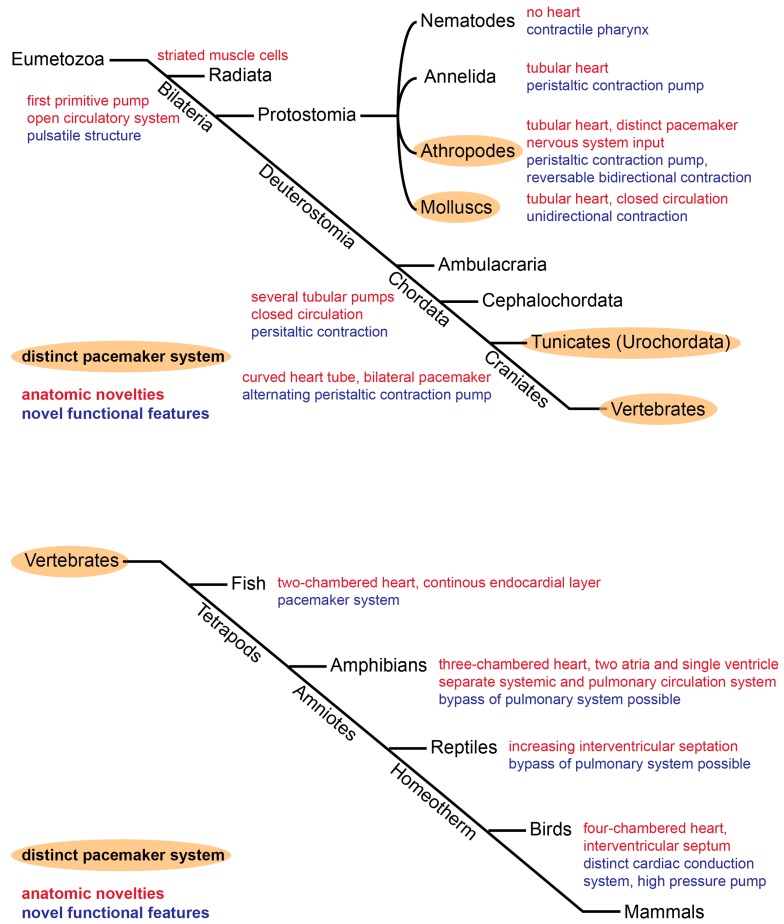
Evolutionary adaption of the cardiac circulation system, with regard to important morphological (red) and functional (purple) novelties. Heart and pacemaker evolution of existent Eumetazoans, from the presumptive common bilaterian ancestor to vertebrates (**top**) and within the vertebrate subphylum (**bottom**). Orange: All groups with an intrinsic pacemaker system, includes all vertebrates.

Even the most basic heart-like structure shares the common crucial feature of all hearts, the ability to rhythmically contract and pump fluid through the body. Thus, the heart is the motor of a fluid-based transport system for nutrients, metabolites, and oxygen. Even animals with radically different lifestyles and body plans, such as insects, fish, birds, and terrestrial animals show a striking conservation in cardiac function [1,7,11,12,13] (Figure 1). The cardiac pacemaker and conduction system in the mammalian heart can be considered as an important advancement to increase cardiac efficiency. Mammals possess a sophisticated network of pacemaker nodes, specially coupled cardiomyocytes and a fast conduction system enabling coordinated, sequential contraction of the chambered heart. In comparison, the primitive tubular pumps in invertebrates resemble early mammalian embryonic hearts both in structure (slow-conducting, poorly coupled myocytes, lack of valves and a conduction system) and function (peristaltic contraction pattern) [2,6,12,14,15,16,17]. It is appealing to hypothesise that these analogies reflect the ancestral background of the mammalian heart. Thus, analysis of heart morphogenesis from an evolutionary perspective might help to understand the mechanisms observed during embryonic development. Many of the morphological changes of the heart have been attributed to physiological adaption of an ancestral cardiac network to an increase in metabolic rate and body size and complexity, and the transition from aquatic to terrestrial habitats. With regard to the pacemaker, it is unclear when exactly the distinct structures evolved.

Pacemaker cells are highly specialized myocardial cells whose intrinsic ability to rhythmically depolarise and initiate an action potential is responsible for the basal heart rate. They are located in the sinoatrial node (SAN) in mammals and the corresponding structures in other vertebrates and several invertebrates (Figure 1). The capacity to trigger an action potential without external stimulation distinguishes pacemaker cells from the surrounding working myocardium. There have long been two hypotheses addressing the mechanism behind the pacemaker capacity. On the one hand is the expression of Hcn4, a specialized ion channel allowing Na^+^/K^+^ ion influx (*I_f_*) in response to hyperpolarisation specifically in pacemaker cells [18,19,20]. On the other hand is an oscillatory release of Ca^2+^ from the sarcoplasmic reticulum (Ca^2+^-clock) [21]. However, it has recently been proposed that both hypotheses are correct and cooperate in facilitating the rhythmic depolarization [22,23]. Pacemaker cells are directly coupled to each other as well as to the adjacent working myocardial cells by gap junctions. These allow the exchange of ions from cell to cell, propagating the action potential from the pacemaker cells through the entire myocardium. Gap junctions consist of connexins, transmembrane proteins with different conductive properties [24]. The fast conducting subunits Cx43 and Cx40 are the main connexins expressed in the chamber working myocardium. Pacemaker cells express slow-conducting connexins, Cx45 and Cx32 [25,26]. This ensures the unidirectional propagation of the electrical signal from the pacemaker cells to the working myocardial cells. Congenital or degenerative defects in pacemaker cell function can cause sinus node dysfunction (SND), a major reason for artificial pacemaker implantation [27,28,29].

Regarding the evolution of the cardiac pacemaker system, several questions arise. Did the distinct pacemaker evolve out of necessity to accommodate the increasing morphological complexity of the heart in vertebrates to ensure a controlled contraction pattern? Did it evolve to ensure coordinated, unidirectional blood flow in a separated systemic-pulmonary circuit? Was it crucial as a mediator to allow heart regulation by the nervous system?

## 2. Origin of the Basic Tubular Heart

The cardiac pacemaker might be described as the specialised, intrinsic structure initiating the cardiac muscular contractions. The first heart-like organ is believed to have evolved in an ancestral bilaterian about 500 million years ago (Figure 1) [1,8,11]. This ancestral “heart” was likely a simple tubular structure, consisting of a single layer of pulsatile cells to force fluid through pericellular interstices without an enclosed vascular system [1]. The initial appearance of muscle-like cells is not entirely clear, but is proposed to have emerged from the gastrodermis prior to the divergence of Cnidarians and Ctenophora from bilaterians [9,30]. Muscle cells are of mesodermal origin and present in all triploblastic animals. Mesodermal cells specifying into early primitive myocytes arose first in bilaterians [1]. It remains to be determined at what stage a subset of cells became functionally dominant to coordinate the cellular contractions. Morphologically, it might have resembled the simple tubular heart found in *Amphioxus* [31].

## 3. Bilateral Pacemaker System in Drosophila Melanogaster

Despite the large evolutionary distance between arthropods and mammals, there is compelling evidence supporting a close structural relationship between cardiac systems. This indicates that a basic tubular heart has been present in the common bilaterian ancestor. Although gene regulatory modifications to accommodate the growing organism lead to morphologically distinct structures, common basic mechanisms are still conserved [1].

Heart formation in arthropods has been widely studied in the most prominent model organism, the fruit fly *Drosophila melanogaster*. The drosophila heart (also referred to as the dorsal vessel) is a tubular organ consisting of a single layer of contractile mesodermal cardioblasts and an overlying pericardial cell layer [1,16]. As generally found in arthropods, drosophila has an open circulatory system with a dorsally positioned heart able to pump haemolymph through the body [32]. The heart functions as a linear peristaltic pump and develops in several repetitive segments [16,33]. There are no distinct chambers, but an aortic valve structure at the anterior opening supports fluid flow direction [1,9,32]. On the posterior side, there are three (larval) or four (adult) pairs of ostia with a valve-like structure [16,32,34,35]. Ostia are thought to be the drosophila analogues to vertebrate inflow tract structures. Their formation depends on a distinct gene expression programme, similar to the differential development of sinus venosus structures versus chamber myocardium in vertebrates [35]. The primary pacemaker is situated at the posterior end of the heart and peristaltic contractions move anteriorly to expel haemolymph into the aorta [36]. At the anterior side a secondary site of contraction initiation has been observed. This allows for a reversal in haemolymph flow [34].

Molecularly, the cardiac muscle cells in drosophila and mammalian species show a striking degree of similarity. Transcription factor *tinman*, the drosophila homologue of *Nkx2.5*, is the determining factor underlying cardiomyocyte differentiation [32,37,38]. Its expression is observed in all cardiac cells. Notably, a subset of cardioblast at the posterior portion of the cardiac vessel lack *tinman* expression [39]. *Seven*-*up*, the homologue of vertebrate *NR2F1 and -2* (Coup-TFI, -TFII), is specifically expressed in the posterior part of the dorsal vessel [32,35,40]. These cells will form the ostia and can be distinguished by re-expression of *dorsocross* genes (Tbx) and expression of *wingless* (Wnt1) [35,39,41]. Furthermore, homologues of important mammalian cardiac factors also partaking in drosophila cardiogenesis are *tailup* (Isl1), *pannier* (Gata4), *mid* (Tbx20), and *Dpp* (Bmp2) [41,42,43,44,45].

Since larvae lack nervous innervation of the heart, only an intrinsic pacemaker potential can initiate the peristaltic contractions [46,47,48]. After metamorphosis, the heart is innervated and receives neuronal input [48,49]. It therefore combines two established mechanisms of rhythmic contraction, external input from the central nervous system and intrinsic control by an independent myogenic pacemaker. Pharmacological studies showed that the important ion-channels found in mammalian myocytes are also present in drosophila [50]. The only major difference was the substitution of the inward sodium current with an inward calcium current as the main depolarization current [32,50]. The mechanism underlying the pacemaker potential in drosophila has not been identified. Drosophila melanogaster I_h_ (DMIH), a homolog of the pacemaker-specific hyperpolarization activated cyclic nucleotide gated potassium channel 4 (HCN4) is present. It similarly encodes a subunit of the slow inward hyperpolarisation-activated potassium channel (I_h_-channels) [51]. However, whether I_h_- is present in the drosophila pacemaker remains to be determined. The *Ork1* gene, encoding a two-pore domain potassium channel facilitating an open rectifier K^+^-current, is a critical component of the drosophila pacemaker system. Ork1 regulates the duration of the slow diastolic depolarisation without influencing basal cardiac automaticity [52]. Whether pacemaker depolarisation in drosophila relies on a mechanism similar to the funny current *I_f_* or a calcium clock mechanism as described in mammals remains to be determined.

## 4. Basic Circulation System in Early Deuterostomia

A basic, but well-studied organism is the tunicate *Ciona intestinalis* (ascidia, urochordata). *C. intestinalis* has an open circulatory system with a curved, V-shaped heart [53]. The tube consists of cardiac myoepithelium containing striated myofilaments and an outer pericardial lining, but lacks an endocardial cell layer [54]. Deuterostome evolution coincided with a multiplication and functional divergence of contractile proteins [1]. There are no chambers or valves discernible and the tube itself does not show morphological polarity [11]. The heart tube is situated on the ventral side of the body, close to the stomach. It opens into single vessels at the posterior end connecting to the endostyle. At the anterior end, it is connected to the dorsal part of the pharynx [54,55]. A series of studies by Kriebel et al. in the 1960s morphologically and physiologically characterised the pacemaker system in tunicates [56,57,58,59,60]. Early electrophysiology and microscopy studies could localise two independent myogenic pacemakers, one at the posterior and one at the anterior opening of the ciona heart tube generating rhythmic peristaltic contractions [1,55]. It is unclear whether one of the pacemakers has a dominant function. The contractions show an alternating pattern and velocity is independent of direction [56,58,61]. The reversal of the pumping direction might be a compensating mechanism for inefficiency of the unidirectional fluid flow or a reaction to external stimuli [11,13,55,58,62]. Nervous system innervation of the heart appears to be absent [58,63]. Humoral modulation and pharmacological alterations of pacemaker frequencies have been reported. However, whether a similar mechanism of control as in higher vertebrates exists remains unclear [60,64].

Several factors important during mammalian heart development are also conserved in ciona, which possesses orthologues of NK homeobox (NKX), GATA binding protein (GATA), T-box (TBX), and heart and neural crest derivatives expressed (HAND) factors [62,65]. Recently, Stolfi et al. identified *Islet*-expressing migratory cells in developing ciona embryos, which follow mechanisms homologous to the second heart field (SHF) in vertebrates [66]. However, these cells did not contribute to the heart, but to the adjacent pharyngeal structure. It can be speculated that further evolutionary progress in cardiac differentiation factors lead to a reallocation of *Isl*^+^ cells [66]. This indicates that an *Islet*-expressing precursor population is highly conserved and might have already been present in the early bilaterian ancestor. Evidence for a distinct cardiac conduction system has not been found in the ciona heart. Electrical coupling is believed to be facilitated by tight junctions between adjacent myocardial cells without a preferred conduction pathway or direction [56,57,58,62].

## 5. Transition to a Sequential Contraction Pattern in Lower Vertebrates

The evolutionary step from invertebrates to vertebrates includes significant remodelling of the cardiac and circulatory system. The transition from a linear tube to a chambered heart is still not fully understood. Therefore, it is also not clear whether the positioning and function of the primary pacemaker in the complex vertebrate heart is a vertebrate-specific evolutionary novelty or secondary to the major morphological remodelling of the heart tube.

With the evolution of the early chordates came a rapid structural and functional diversification of the cardiac system, such as a looped heart with separate chambers, functional valves, and trabeculae, facilitating unidirectional blood flow. In higher vertebrates, heart function is eventually controlled by a coordinated pacemaker and conduction system [1,7,8,9,11,61,67].

All vertebrates have a closed circulatory system with an endocardial layer lining the heart [11]. This also abolishes the possibility to supply the myocardium by direct perfusion. Instead, an epicardial layer and coronary artery system is established to serve the thickening myocardial layer [11]. A basic configuration of alternating slow-conducting and poorly contracting pacemaker components with fast-conducting myocardium appears to be conserved in all higher vertebrates with multi-chambered hearts [68].

## 6. Two-Chambered Heart in Zebrafish

**Figure 2:**
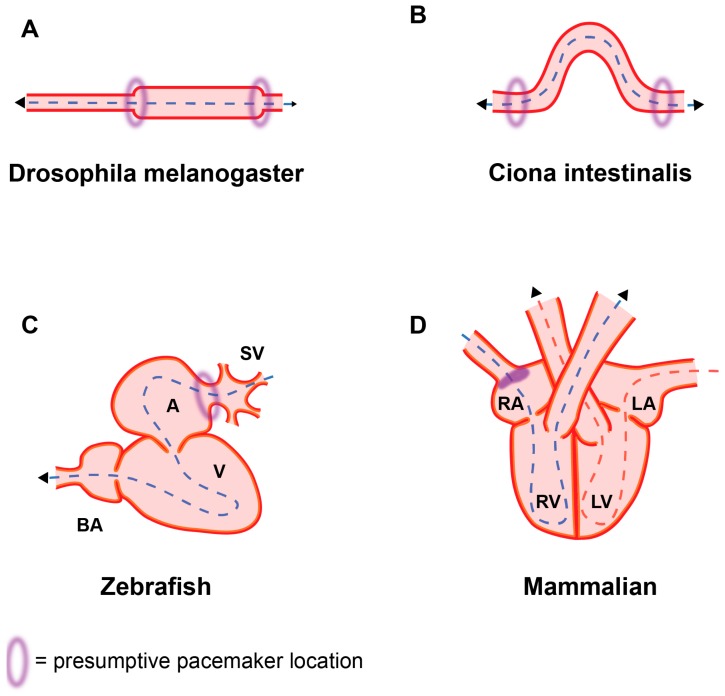
Illustration of heart evolution. (**A**) Drosophila dorsal vessel and (**B**) Ciona heart with bilateral pacemaker structures. (**C**) two-chambered zebrafish heart with pacemaker ring at sinoatrial junction. (**D**) Four-chambered mammalian heart, primary pacemaker in the sinoatrial node (SAN). (**A**–**C**: anterior on the left, posterior on the right), Arrows indicate direction of blood flow. Red = myocardial/muscle layer; orange = endocardium; A = atrium; V = ventricle; SV = sinus venosus; BA = bulbus arteriosus.

Fish have a single circuit circulation system. The heart is positioned upstream of the gills, rendering a single pumping system sufficient. The best-studied representative is the teleost zebrafish (*Danio rerio*) [69,70]. The adult zebrafish heart consists of two contractile chambers, a single atrium, and a single ventricle delimited by valves at the atrioventricular junction. The ventricle of mature zebrafish hearts is a thick muscular pump and is highly trabeculated. Furthermore, it has an enlarged outflow tract, the bulbus arteriosus, and a non-muscular inflow reservoir, the sinus venosus (Figure 2) [71,72].

The onset of myocardial contractions is observed early during embryonic development, shortly after formation of the linear heart tube. It originates from the venous pole and initially has a peristaltic contraction pattern [70]. The embryonic fish heart resembles the dynamic suction pump mechanism seen in invertebrate hearts [73]. During cardiac looping, the onset of a conduction delay at the atrioventricular (AV) canal leads to a sequential atrial-ventricular contraction pattern [74]. An optogenetic study localised the functional pacemaker in the embryonic zebrafish heart at the inner curvature of the atrium, immediately adjacent to the venous pole and restricted to a small number of cells [75]. Voltage dynamics visualisation showed that depolarisation originates from the sinoatrial (SA) region [74,76]. This location is considered to be similar to the SAN in mammalian hearts. Interestingly, temperature acclimation studies in another teleostei, the rainbow trout, also located the primary pacemaker at the sinoatrial junction [77].

Information on the molecular regulation of the pacemaker domain in the zebrafish has long been sparse, largely due to the lack of a pacemaker specific marker. The LIM-homeodomain transcription factor Islet-1 (Isl1), an important factor in the development of cardiomyocyte precursors of the second heart field, was identified as the first pacemaker specific molecular marker [78]. Zebrafish embryos lacking Isl1 display a bradyarrhythmic and progressive sinus block phenotype reminiscent of pacemaker function defects [78,79]. Isl1 expression marks cardiac pacemaker cells from 48 h post fertilisation (hpf) to adulthood. Its expression remains highly restricted to a small number of cardiomyocytes at the sinoatrial junction. The cardiac pacemaker cell domain in zebrafish is organised in a ring structure around the sinoatrial junction, rather than a compact node [78]. The initiation of depolarisation at the inner curvature side of the sinoatrial junction might reflect an intrinsic hierarchy between the pacemaker cells in the ring with the dominant cells dictating the heart rhythm. Electrophysiological analyses of isolated adult Isl1-expressing cardiomyocytes show the characteristic pacemaker cell properties such as spontaneous independent depolarisation [78]. Furthermore, the adult pacemaker domain shows expression of *hcn4*, *tbx2b*, and *bmp4*, and is devoid of *nppa* expression [78,80]. Bmp signalling is essential for atrial formation and inhibits cardiomyocyte differentiation [81,82]. Bmp4 is acting downstream of Isl1 since the expression of *bmp4* is lost specifically at the sinoatrial junction of Isl1 knockout mutant embryos [79].

Shox2 is expressed in the embryonic pacemaker domain in zebrafish. Antisense morpholino knock-down of *shox2* in zebrafish embryos results in bradycardia suggesting that Shox2 plays a role in SAN development in fish, as it does in mice [83]. Both the zebrafish and human genome contain *shox*, a homologue of *shox2*. In Human, *SHOX* and *SHOX2* are very similar in sequence, have a common homeodomain and appear to be redundant in function [84]. In zebrafish, *shox* expression has been identified in the putative heart [85]. The absence of *Shox* in the mouse genome might explain the severe developmental cardiovascular defects in *Shox2*^−/−^ mice.

In the AV canal of the adult zebrafish heart, a secondary pacemaker has been identified. After resection of the atrium, the pacemaker activity in the AV canal is sufficient to initiate ventricular contraction [86]. In the developing heart, the T-box transcription factor Tbx2b is expressed in the AV canal, whereas the working myocardial marker Nppa is expressed in the chamber myocardium and is absent from the AV canal [87]. The mature electrophysiological properties and electrocardiography (ECG) pattern in zebrafish are comparable to the mammalian heart [72,88,89,90]. The important ion currents for depolarisation, plateau phase, and repolarisation (*I_Na_*, *I_Ca,L_* and *I_Kr_*) are similarly distributed as in mammalian hearts. *I_Ca,T_*, which has a prominent role in mammalian pacemaker cell depolarisation was expressed in all cardiomyocytes of the zebrafish heart. Unlike the mammalian situation, expression persists in mature cardiomyocytes [89].

In mammals, molecular and electrophysiological properties of pacemaker cells have been characterised extensively. The expression of a hyperpolarisation-activated slow rectifier potassium channel is considered the inherent property of mammalian pacemaker cells. Characterisation studies of the zebrafish mutant *slow-mo* argued for the existence of a hyperpolarisation-activated inward potassium current *I_h_*. The mutant presents with bradycardia persisting into adulthood [91,92,93]. However, unlike the funny current (*I_f_*) in mammalian pacemakers, *I_h_* was found in all cardiomyocytes and the underlying genetic components have not been identified, rendering it unsuitable as a pacemaker cell marker [91,92].

Expression data for homologues of the mammalian cardiac connexins (Cx30.2, Cx40, Cx43, Cx45) is sparse in zebrafish [94]. *Cx43* (homologue of Cx43) expression has been observed in the embryonic heart [74,95]. *Cx45.6* (homologue of Cx40) is expressed in the chamber myocardium of the ventricle and atrium, similar to the mammalian expression pattern [74,96]. Both pacemaker-specific mammalian connexins (Cx30.2, Cx45) have not been described in zebrafish so far.

The adult zebrafish heart shows profound neuronal innervation, including innervation of the SA junction near *hcn4* and *isl1* expressing pacemaker cells. The neuronal plexus at the SA junction contains cholinergic, adrenergic, and nitrergic axons [80]. Furthermore, zebrafish embryonic hearts express muscarinic acetyl choline and β-adrenergic receptors and the cholinergic agonist carbachol causes a decrease in heart rate (i.e., bradycardia) [86,97]. This indicates a neuronal control of the pacemaker domain possibly similar to the neuronal crosstalk reported in the mammalian SAN [80].

## 7. Septation and Conduction System Development in Amphibians

Contrary to zebrafish, amphibians and reptiles are not well studied on a molecular level. Gene expression information remains sparse. A common amphibian model organism is the clawed frog *Xenopus laevis* [98]. Heart rate measurements in Xenopus larvae [99] and adults [100] showed that the ECG pattern is comparable to mammals and contractions showed sequential atrial-ventricular contraction with a delay at the AV junction. Furthermore, drug sensitivity studies showed that the pacemaking system in Xenopus interacts with sympathetic and parasympathetic nervous system input [99]. The location of the primary pacemaker was not addressed in this study. It remains unclear whether this pacemaker shows a similar right-sided laterality as the mammalian SAN.

As described for zebrafish, the presence of an organised ventricular conduction system in Xenopus is unclear. Due to the absence of a ventricular septum, it has been hypothesised, that the ventricular trabeculae or discrete conduction pathways in the ventricular wall might constitute a fast pathway to the apex of the ventricle [71,101].

The reptilian heart on the other hand shows a varied degree of ventricular septation [102,103,104]. The sinus venosus is well developed and the first reservoir to receive blood from the venous system and has been considered to be a fourth chamber [103,105,106].

Data on the embryonic development of reptilian hearts is sparse. Early morphological development is similar to that of higher vertebrates [67,107,108]. An initially peristaltic contraction pattern of the early heart tube has been observed in several reptilian species [107]. Cardiac contractions in the mature reptile heart originate from the sinus venosus. They result in a characteristic SV wave in ECG measurements that precedes the P wave (atrial depolarisation) [106]. The pacemaker might be less compact than in mammals, leading to a widespread pacemaker area across the sinus venosus, leading to its referral as a pacemaker chamber [109]. The fully septated, four-chambered crocodilian heart morphologically closely resembles the avian and mammalian heart. A SAN with spontaneously depolarising pacemaker cells has been morphologically outlined in the crocodile heart [12,110].

Nervous system innervation of the heart has been described for various reptiles and amphibians [111,112,113,114]. Whether these directly interact with the pacemaker structure could not be clarified yet, due to a lack of understanding of the pacemaker system in these animals.

## 8. Complex Pacemaker and Cardiac Conduction System in Birds and Mammals

The earliest electrophysiological observations of the primary action potential initiation in the developing chick heart has located the pacemaker site at the left posterior inflow tract of the heart at Hamburger and Hamilton stage 10 (HH10). During the course of heart morphogenesis, a notable shift of the primary pacemaker to the ventral surface of the right inflow tract is accompanied by a RhoA-dependent spatial restriction of a Tbx18^+^ Isl1^+^ Hcn4^+^, and Nkx2.5^−^ myocardial subpopulation [115,116]. Electrophysiological studies demonstrated impulse initiation in the right atrium (HH29) [117]. Through direct cell labelling, Bressan et. al identified a distinct population of pacemaker precursor cells within the right lateral plate mesoderm of the developing chick embryo, favouring a very early pacemaker cell specification [115].

## 9. Transcription Factor Network Patterns the Mammalian SAN

The primary pacemaker in the mammalian heart is located in the SAN in the dorsal wall of the right atrium, at the junction with the superior vena cava [118]. The pacemaker cells in the SAN are automatic and the intercellular conduction velocity is slow. The SAN is surrounded by connective tissue and has a nodal artery [119].

In mice, the SAN develops within the right sinus horn of the sinus venosus from E9.5 onward, and can be morphologically identified at E10.5 [120]. Until E9, the transcription factor *Nkx2.5* is expressed in all cardiac cells of the primary heart tube [121,122]. Initially, *Hcn4* is expressed in the early heart tube, highest in the *Nkx2.5^+^* inflow tract [123,124]. Between E9 and E12, *Tbx18^+^ Nkx2.5^−^* progenitor cells differentiate into myocardium, are added to the inflow tract, and form the sinus venosus [122]. *Hcn4* is highly expressed in the *Tbx18^+^ Nkx2.5^−^* sinus venosus cells and becomes downregulated in the *Nkx2.5^+^* cells of the inflow tract, which have moved on to form the *Cx40^+^* and *Nppa^+^* atrial myocardium [123,125]. A subpopulation of right-sided sinus venosus cells maintains *Isl1* expression and has initiated *Tbx3* expression upon their differentiation [123,126]. These cells most likely represent the SAN primordium, and form a thickening within the sinus venosus immediately adjacent to the *Cx40^+^* atrial cardiomyocytes. *Pitx2c* controls right-sided SAN development by suppressing SAN development within its own expression domain at the left side of the atrium and sinus venosus. *Pitx2c* null mice develop a second left-sided SAN primordium as part of right atrial/sinus venosus isomerism [123,127].

With further enlargement of the sinus venosus (now comprising the venous side of the venous valves and the common, right, and left sinus horns), the *Nkx2.5^−^ Cx40^−^ Isl1^+^ Tbx3^+^ Hcn4^+^* SAN primordium runs from the thickening in the superior caval vein (*Tbx18^+^*; called ‘head’) to the proximal part of the right venous valve (*Tbx18^+^* derived; called ‘tail’) [125]. During the first stages of development, the size of the SAN mainly increases by addition of cells from the progenitor pool. However, SAN cells divide slowly, which will also contribute to growth of the SAN. During early foetal stages, Hcn4 expression is maintained in the SAN but is downregulated in the remaining cells of the sinus venosus. Cx40 and Cx43, not expressed in the SAN, are upregulated in the remainder of the sinus venosus (referred to as atrialisation of the sinus venosus) [123].

*Nkx2.5^ires-Cre,+^*; *R26R^lacZ^* lineage tracing confirmed that the SAN forms from Nkx2.5^−^ cells [123]. Nevertheless, Nkx2.5 is briefly expressed in sinus venosus precursors but is turned off prior to their differentiation [128]. *Tbx18* was found to be expressed exclusively in the *Nkx2.5^−^* sinus venosus, a pattern that is conserved in human, mouse, xenopus, chicken, and zebrafish [122,129]. Genetic lineage tracing using *Tbx18^cre,+^*/*R26R^lacZ^* mice revealed that the entire sinus venosus, including the SAN head and tail, are derived from *Tbx18^+^* precursor cells, although the expression of *Tbx18* becomes downregulated in the tail part during development [125]. The critical role of *Tbx18* in SAN and sinus venosus formation was shown in *Tbx18*^−/−^ mice, that form a hypoplastic sinus venosus and SAN head region. In contrast to morphological abnormalities, the small SAN that is formed in *Tbx18^−/−^* mice does not exhibit an altered SAN gene programme or changes in heart rhythm. However, transduced overexpression of *Tbx18* in neonatal ventricular cardiomyocytes in vitro and in vivo was sufficient to induce a SAN-specific phenotype and spontaneous depolarization [130]. Moreover, injections with *Tbx18* expressing adenoviral vectors in pig ventricles with complete heart block showed enhanced heart rhythm and decreased expression of *Nkx2.5* and *Cx43* in the injection area, whereas *Hcn4* levels were upregulated. Electroanatomic mapping further showed increased pacemaker activity at the injection site [131]. In contrast, transgenic misexpression of *Tbx18* in the mouse heart did not result in ectopic pacemaker cell formation [132]. Therefore, the extent of *Tbx18* mediated “reprogramming” and the precise mechanism needs to be addressed further.

*Tbx3* in the SAN acts as a transcriptional repressor of genes of the working myocardial gene programme, including *Nppa*, *Nppb*, *Scn5a*, *Cx40*, and *Cx43*. In addition, it activates conduction system genes like *Hcn4.* It was shown that ectopic expression of *Tbx3* in developing atrial myocardium reprograms these cells into bona fide functional pacemaker cardiomyocytes [133,134]. This indicates that *Tbx3* functions as a molecular switch between the genetic programme of working myocardium and SAN.

In the heart tube, *Tbx3* is repressed by *Nkx2.5*. *Nkx2.5^−/−^* embryos die before E10 due to abnormal heart looping and show increased expression of *Tbx3* and *Hcn4* in the heart tube [123]. Ectopic activation of *Nkx2.5* in SAN cells induces expression of working myocardial markers *Nppa* and *Cx40* and repression of *Hcn4*, indicating that *Nkx2.5* activates the working myocardial gene programme and suppresses the pacemaker programme [135]. The homeodomain transcription factor *Shox2* is expressed in the SAN and sinus venosus and was shown to repress *Nkx2.5* and control expression of *Bmp4* [135,136,137]. *Shox2*^−/−^ embryos have a reduced SAN size and exhibit increased expression of *Nkx2.5*, *Cx40*, and *Cx43*, and decreased expression of *Hcn4*, *Tbx3*, and *Isl1* in the SAN primordium and show cardiac arrhythmias and embryonic lethality at day E11.5 [138]. These studies indicate a regulatory function of *Shox2* in repressing the working myocardial programme via the repression of *Nkx2.5* and induction of Tbx3. Expression of both *Tbx3* and *Shox2* depends on Tbx5 [139], a core cardiac transcription factor expressed from early stages in the cardiac progenitors. Shox2 acts upstream of *Isl1* in pacemaker specification [83]. *Isl1^−/−^* mice exhibit severe abnormalities with respect to second heart field formation [140] but conditional *Isl1* knock-outs in the Hcn4 expression domain show downregulation of *Tbx3*, *Shox2*, *Hcn4*, *Bmp4*, and *Cacna1g* in the developing SAN, whereas *Nppa*, *Gja1*, *Gja5*, and *Scn5a* were upregulated [126,141]. Together with the observation that Isl1 is essential for functional pacemaker development in zebrafish, this implies an important role for Isl1 in activating the mammalian pacemaker programme. Interestingly, ISL1 is expressed in the cells of the human SAN of both the embryonic and adult heart suggesting an evolutionary conserved function [17,78,142,143].

In both zebrafish and mice, Bmp signalling is involved in atrioventricular canal (AVC) and atrioventricular conduction system development [144,145,146]. Bmp signalling co-operates through Smads with Gata4 and histone acetylases (HATs) and histone deacetylases (HDACs) to activate AVC-specific enhancers in the AVC and inactivate them in the atrial and ventricular chamber myocardium [145]. The correlation between the HDAC inhibition and conduction system development is further emphasized by knockout experiments, where deletion of *HDAC1* and *HDAC2* in mice resulted in neonatal lethality, bradycardia and increased expression of *Cacna2d2*, a calcium channel that is present in the conduction system [147]. Furthermore, an enhancer region in the first intron of the *Hcn4* locus is activated in the ventricular myocardium after exposure to both transcription factor *Mef2c* and *HDAC* inhibition [148]. Therefore, it is tempting to speculate that SAN gene expression is regulated by a mechanism involving BMP-signalling, core cardiac transcription factors, and HDAC activity.

## 10. Conclusions

**Figure 3:**
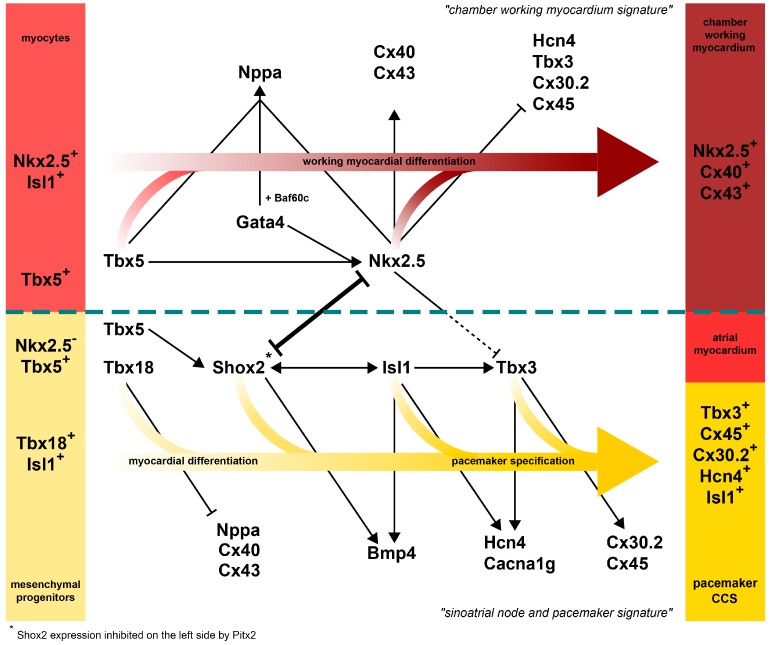
Important factors in the specification of pacemaker cells in the SAN and atrial working myocardium. SAN cells arise from a *Tbx18*^+^ *Nkx2.5*^−^ mesenchymal progenitor population located adjacent to the *Nkx2.5*^+^ posterior heart tube myocytes. Tbx18 is the main driving factors of myocardial differentiation in the mesenchymal progenitors. It delineates the SAN primordium by competing with Tbx5 and functionally repressing atrial differentiation factors such as Gata4, Nkx2.5, and Nppa. Shox2 inhibits Nkx2.5 expression, activates Tbx3 and interacts with Isl1. Shox2 is a direct target of laterality factor Pitx2 and is inhibited in the left compartments of the developing heart. Tbx3 is the main factor to directly or indirectly activate pacemaker-specific factors. Tbx5 interacts with Gata4 and Nkx2.5 to initiate working myocardial cell differentiation. Tbx5 represses Shox2 in the working myocardium. Nkx2.5 is the main determining factor for chamber myocardial cells and activates working myocardium-specific factors. The transcription factor network leads to the establishment of specific gene expression signatures. The SAN is characterised by the high expression of Tbx3, Shox2, Isl1, Bmp4, Hcn4, Cacna1g, Cx30.2 (mouse), and Cx45, corresponding with the low expression or absence of Cx40, Cx43, and Scn5a in embryos and adults. The working myocardium shows a contrary expression pattern, with the high expression of Cx40, Cx43, Scn5a, and Nppa corresponding to low or absent expression of Cx30.2 (mouse), Cx45, and Hcn4.

The coordinated rhythmic contraction is the fundamental principle of cardiac function. Specialized cardiac pacemaker cells are responsible for initiating the electrical impulse. Despite the vast morphological differences between the simple invertebrate heart and the structurally more complex mammalian heart, there is a striking degree of evolutionary conservation of the fundamental functional and molecular pathways. The presence of specialized pacemaker cells at the inflow pole of the heart is the common feature of all cardiac systems. In primitive invertebrate species, pacemaker cells are present at several locations, even allowing a reversal of the pumping direction. In contrast, the closed circulatory systems of vertebrates rely on a strictly unidirectional blood flow driven by a single dominant pacemaker structure (Figure 2). When and how this restriction to a single dominant pacemaker structure occurred during evolution remains unclear. During cardiac differentiation, the precursor cells and derived pacemaker cardiomyocytes execute a cascade of patterning factors, driving a pacemaker-specific gene programme. The pacemaker cells remain embedded in and coupled to the surrounding myocardium, requiring a highly-controlled delineation from the working myocardial gene programme (Figure 3). This highlights an ancestral network of gene regulation, which does not seem to have changed dramatically as the heart evolved from a simple suction pump to the complex four-chambered heart in mammals.

The zebrafish is increasingly being used as a model to address pacemaker development and function. Considerable homology between zebrafish and mammalian heart development and physiology and the feasibility of high-throughput genetic or pharmacological manipulation provide promising opportunities for cardiac research. Furthermore, novel gene editing techniques such as the Transcription activator-like effector nucleases (TALEN) and Clustered regularly interspaced short palindromic repeats (CRISPR-Cas9) systems allow for the precise modelling of deleterious human gene mutations [69,70,89,149,150,151,152].
